# Odd- and Branched-Chain Fatty Acids in Lamb Meat as Potential Indicators of Fattening Diet Characteristics

**DOI:** 10.3390/foods10010077

**Published:** 2021-01-03

**Authors:** Pilar Gómez-Cortés, Francisco Requena Domenech, Marta Correro Rueda, Miguel Ángel de la Fuente, Achille Schiavone, Andrés L. Martínez Marín

**Affiliations:** 1 Instituto de Investigación en Ciencias de la Alimentación (CSIC-UAM), Universidad Autónoma de Madrid, Nicolás Cabrera 9, 28049 Madrid, Spain; p.g.cortes@csic.es (P.G.-C.); mafl@if.csic.es (M.Á.d.l.F.); 2 Departamento de Biología Celular, Fisiología e Inmunología, Universidad de Córdoba, Ctra. Madrid-Cádiz km 396, 14071 Córdoba, Spain; v02redof@uco.es; 3 Departamento de Producción Animal, Universidad de Córdoba, Ctra. Madrid-Cádiz km 396, 14071 Córdoba, Spain; martacorrero@gmail.com; 4 Dipartimento di Scienze Veterinarie, Università degli studi di Torino, 10124 Torino, Italy; achille.schiavone@unito.it

**Keywords:** fatty acids, meat, lambs, feeding, discriminant analysis

## Abstract

There is a growing interest of researchers in meat authentication in terms of geographical and dietary background of animals, and several analytical methods have been proposed for the purpose of investigating this. We hypothesized that the odd- and branched-chain fatty acid (OBCFA) profile in intramuscular fat (IMF) might suffice to distinguish lamb meat entering the food chain supply on the basis of the type of diet fed to lambs during the fattening period. A total of 30 individual OBCFA profiles, quantified by gas chromatography, of IMF of Manchego lambs were used. During the fattening period (42 days), the lambs were fed three diets differing in concentrate composition: (i) Control, concentrate typical of commercial fattening rations, rich in starch and based on cereals and soybean meal; (ii) Camelina, similar to Control but replacing 50% of the soybean meal with camelina meal; and (iii) Fibrous, concentrate rich in neutral detergent fiber (NDF), based on fibrous by-products and not including cereals nor soybean meal. The OBCFA were grouped into three classes (linear odd, iso and anteiso fatty acids) and were then submitted to a linear discriminant analysis, using the feeding treatments as grouping variable and the OBCFA class contents in IMF as quantitative variables. The results suggested that a high NDF to starch ratio of the concentrate, being the lowest for Control (CON) treatment and the highest for Fibrous (FIB) treatment, would be negatively related to the odd/anteiso ratio and positively related to the iso/(anteiso+odd) FA ratio in IMF. Determination of OBCFA profile in lamb meat would be useful to monitor the feeding regime (starch- or NDF-rich) of lambs entering the food chain supply.

## 1. Introduction

Europe has very different sheep production systems in the Northern and Mediterranean areas as feeding and husbandry are adapted to local environmental conditions and agricultural practices. Moreover, there are great differences in the acceptability of lamb meat by consumers across regions due to flavor variations [1], which can be directly ascribed to the feeding background of the lambs [2]. Again, consumers are concerned about information about sheep production systems and, specifically, the type of diet the sheep are fed because they are aware of its effects on lamb meat quality [3]. In this regard, there has been a growing research interest in meat authentication in terms of the geographical and dietary background of animals, and several analytical methods have been proposed for that purpose [4]. Those methods are based on more or less complicated laboratory techniques intended to identify reliable predictors such as fatty acids (FA), volatile compounds, stable isotopes or a variety of metabolites in meat [5,6,7,8,9]. The FA profile of intramuscular fat (IMF) has proven to efficiently discriminate between diets fed to fattening lambs [5]. Furthermore, intramuscular FA are directly linked to organoleptic and nutritional attributes of ruminant meat [10,11].

Odd- and branched-chain fatty acids (OBCFA) are quantitatively minor FA that are almost exclusively of ruminant fat. These FA have attracted great attention not only because they may serve as biomarkers of rumen function [12,13] but also because of their putatively beneficial effects on human health [14]. OBCFA found in ruminant fat mainly come from intestinal digestion of bacteria washed out from the rumen in solid and liquid phases [15,16]. In turn, available data show that the contents of OBCFA in the lipids of rumen bacteria differ between species, i.e., cellulolytic bacteria usually contain more iso FA (although some strains are rich in anteiso FA), whereas amylolytic bacteria are mainly rich in linear odd-chain FA, with a few strains displaying high contents of anteiso FA [12,13]. The relative abundance of rumen bacterial populations heavily depends on diet composition [17,18]. Likewise, the relationship between diet characteristics, the relative abundance of rumen bacterial species and the OBCFA profile of rumen contents has been demonstrated [19,20].

Therefore, we hypothesized that OBCFA contents in IMF might suffice to distinguish lamb meat entering the food chain supply on the basis of the characteristics of the diet fed to lambs during the fattening period.

## 2. Materials and Methods

This work was carried out with the data from a study whose results have been published elsewhere [21,22]. Briefly, the animal protocol was fully in compliance with the European Union and Spanish regulations on animal welfare and experimentation. A total of 105 uncastrated male lambs of the Manchega breed with an initial bodyweight (BW) of 13.9 ± 1.74 kg and an age of 35 ± 7 days old were randomly allocated to 15 straw-bedded pens. The pens were randomly allocated to one of three treatments (five replicates per treatment): Control (CON), Camelina (CAM), and Fibrous (FIB). The concentrate of CON treatment was cereal-soybean meal based, which is similar to the concentrates commonly used in the feedlot of light lambs (11.4 MJ metabolizable energy/kg, 15.7% crude protein, as fed). The concentrate of CAM treatment was similar to CON but replaced 50% of soybean meal with solvent-extracted camelina meal (11.3 MJ metabolizable energy/kg, 15.6% crude protein, as fed). Finally, the concentrate of FIB treatment did not contain cereals or soybean meal, included several fibrous by-products and was very rich in neutral detergent fiber (NDF) (9.8 MJ metabolizable energy/kg, 15.8% crude protein, as fed). As a result, the NDF to starch ratio was 0.31, 0.40 and 1.54 in CON, CAM and FIB concentrates, respectively. Barley straw was offered as roughage in all experimental treatments. On day 42 of the trial, two lambs per pen (i.e., 10 lambs from each experimental treatment), with the final BW closest to the average pen BW, were tagged to track their carcasses. Then, all animals were sent to a commercial abattoir for slaughter. Samples of Longissimus thoracis muscle were obtained for intramuscular FA analysis after 6 days of aging at 4 °C. Analyses of IMF were made in duplicate. Firstly, muscle was homogenized with a mixture of chloroform and methanol and BHT added as antioxidant. Dilution with chloroform and water separated the homogenate into two layers. The chloroform layer containing all the lipids was collected, and, after removal of the solvent, the total lipids were preserved in amber vials frozen at −17 °C until derivatization. Fatty acids were derivatized to methyl esters by base-catalyzed methanolysis. The OBCFA were quantified by gas chromatography with an Agilent model 6890 N network system (Palo Alto, CA, USA) equipped with an autoinjector and a flame ionization detector (FID) and fitted with a CP-Sil 88 fused silica capillary column (100 m 0.25 mm i.d., Varian, Middelburg, The Netherlands). Injector and detector temperatures were set to 250 °C. Helium was used as the carrier gas, and samples were injected with a split ratio of 1:100. Initial oven temperature was 45 °C. After 4 min, it was increased at a rate of 13° C/min to 165 °C and held for 35 min. Then, oven temperature was increased to 215 °C at 4 °C/min and maintained for 30 min. Individual FA were identified by comparison with standards distributed by Nu-Chek (Elysian, MN, USA).

Statistical analyses were carried out with SAS University Edition 3.8 (SAS Institute, Cary, NC). OBCFA were grouped into three classes (linear odd, iso and anteiso FA) and the requirements of homoscedasticity, absence of outliers and normality were verified with Box, Grubb and Mardia tests, respectively, at *p* > 0.05; multicollinearity was also discarded (variance inflation factor lower than 1.3). Then a linear discriminant analysis (LDA) was performed with the DISCRIM procedure. LDA is a multivariate statistical technique that can be used to differentiate experimental groups and to determine the meaningful variables that contribute most to such differences [23]. In the analysis, the grouping variable were the feeding treatments, and the quantitative variables were the OBCFA class contents in IMF samples. Wilks’ test, Bartlett’s test, squared Mahalanobis distances and cross-validation of generated Fisher’s functions were obtained by means of MANOVA, CAN, DISTANCE and CROSSVALIDATE options, respectively, included in the DISCRIM procedure. The GLM procedure, using the experimental treatments as the fixed effect and the Tukey’s test for mean separation at *p* < 0.05, was also used to help in explaining the LDA results.

## 3. Results

Final BW (25.8 ± 1.12 kg), average daily gain (290 ± 23.8 g/day) and IMF level (1.25 ± 0.22%) did not differ between treatments (*p* > 0.05), but feed conversion ratio was 23% higher in the FIB treatment (*p* < 0.05) [21,22]. Total contents of linear odd, iso and anteiso FA in the Longissimus thoracis muscle of lambs under the experimental treatments are presented in Table 1. The quantitatively main FA within each class of OBCFA were C17:0 (~ 66% of linear odd FA), C17:0 anteiso (~ 82% of anteiso FA) and C14:0 iso (~ 42% of iso FA).

**Figure 1:**
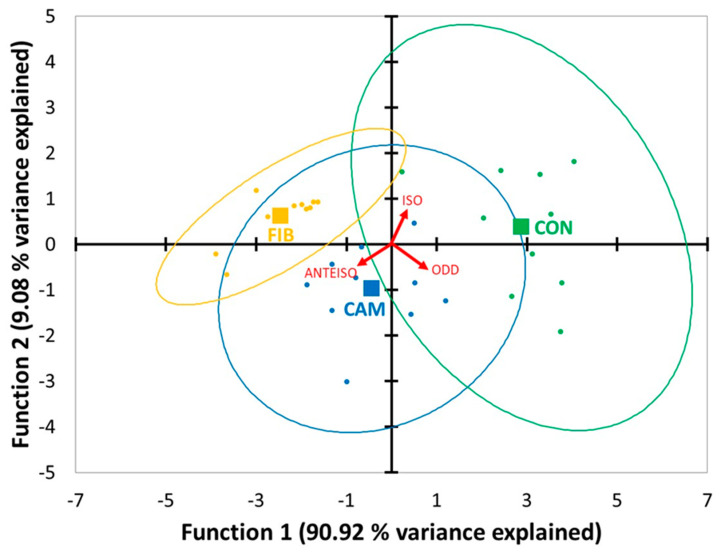
Canonical discriminant plot. Observations correspond to three type of diets: a typical commercial concentrate rich in starch and based on cereals and soybean meal (CON, green dots); a typical commercial concentrate which replaces 50% of soybean meal with camelina meal (CAM, blue dots); and a concentrate rich in neutral detergent fiber based on fibrous by-products and not including cereals or soybean meal (FIB, yellow dots). Centroids are indicated by squares of the corresponding color. Variable correlations with the discriminant functions are represented by red arrows.

Results from the canonical discriminant analysis are shown in Table 2. Eigenvalues, which provide information about the relative efficacy of each discriminant function, were significant according to Bartlett’s test. Canonical discriminant functions (DF) explained 90.92% and 9.08% of total variance, respectively. Canonical correlation of the quantitative variables and the grouping variable was higher in DF1. Wilks’ lambda test, which checks how well each function separates cases into groups, supported the validity of the model. Linear odd and anteiso FA showed the greatest discriminating ability and correlation in DF1, while iso FA had a higher contribution to group separation in DF2 (Table 2 and Figure 1). The centroid of CON treatment showed a positive value in both functions, while the centroid of FIB treatment was negative in DF1 and positive in DF2. The centroid of CAM treatment was negative in both functions (Table 2 and Figure 1). The largest distance was between CON and FIB treatments with CAM treatment in an intermediate position.

Fisher’s classification functions (Table 3) incorrectly assigned one observation from the CON to the CAM group (96.7% of the samples were correctly assigned). When cross-validation was carried out, two samples were mislabeled: one CON observation was assigned to the CAM group and one CAM observation was assigned to the FIB group. As a result, cross-validation properly classified 93.3% of lamb meat samples. Finally, variance analysis showed that the odd/anteiso FA ratio clearly differed between the three treatments, while the iso/(anteiso+odd) FA ratio was different for FIB compared to CON and CAM treatments (Table 4).

## 4. Discussion

Comprehensive discussion regarding growth performance, carcass and meat quality traits as well as IMF composition of the lambs can be found elsewhere [21,22]. In summary, experimental animals were homogeneous, maintained under the same housing and management conditions, and the feeding trial was carried out simultaneously in all experimental groups. Since no differences were found in productive traits or intramuscular fat content between treatments, any differences in intramuscular OBCFA profile should only be ascribed to the type of diet fed to each group of lambs.

**Figure 2:**
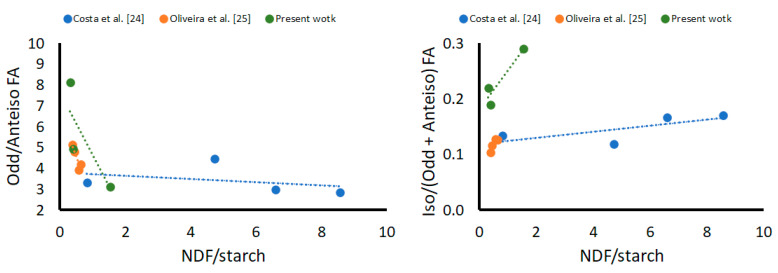
Responses of odd/anteiso and iso/(odd + anteiso) fatty acid (FA) ratios in the intramuscular fat of lambs to the neutral detergent (NDF) to starch ratio of the diet fed during the fattening period.

Our results support those of a recently published research on the validity of the FA profile of meat as a tool to identify the dietary background of lambs by means of discriminant analysis [5] but also indicate that it is not necessary to use an extensive set of FA in IMF for that purpose. The OBCFA contents in IMF suffice to discriminate the meat samples according to the composition of the diet consumed by the lambs during the fattening period. In DF1, odd and anteiso FA (right and left sides, respectively, Figure 1) showed the highest discriminant ability, whereas iso FA (upper-right side, Figure 1) had the highest discriminant ability in DF2. These results suggested that a high NDF to starch ratio of the concentrate, being the lowest for CON treatment and the highest for FIB treatment, would be negatively related to the odd/anteiso FA ratio and positively related to the iso/(anteiso+odd) FA ratio in IMF. This point was further confirmed by the results of the analysis of variance (Table 4). The negative and positive linear responses of the odd/anteiso and iso/(odd+anteiso) FA ratios, respectively, to the increase of the diet NDF to starch ratio that can be noted in Table 4 are in agreement with two papers published in the last few years where lambs were fed fattening diets with intended differences in their NDF/starch ratio [24,25] (Figure 2).

The effects of dietary treatments on ruminal bacteria populations might explain the validity of OBCFA as indicators of the NDF and starch proportions in the fattening diet fed to lambs. Diets rich in NDF raise the abundance of ruminal cellulolytic bacteria in growing lambs [17] without significant differences in meat OBCFA contents when forage is compared with fibrous by-products as an NDF source [26], and cellulolytic bacteria are usually enriched in iso FA, with some strains showing high levels of anteiso FA [12,13]. Moreover, it can be calculated that lambs classified postmortem as high-cellulolytic according to the number of main cellulolytic bacteria identified in the rumen contents had odd/anteiso and iso/(anteiso+odd) FA ratios in their IMF that were ~10% lower and ~17% higher, respectively, than that observed in the lambs classified as low-cellulolytic [27].

Our experimental concentrates did not include extra fat rich in polyunsaturated FA [21]. It is well-known that polyunsaturated FA in the diet had a negative impact on ruminal cellulolytic bacteria populations [28], which might change the OBCFA profile in IMF regardless of the NDF and starch proportions in the diet. In lambs fed the same diet either supplemented or not with linseed during the whole fattening period, the odd/anteiso FA ratio in IMF was ~ 30% lower in the linseed group compared with the control group [29]. Hence, the use of OBCFA profiles in IMF to separate lamb meat samples according to the NDF and starch proportions of the diet fed during the fattening period will not be appropriate when fat sources rich in polyunsaturated fatty acids are supplemented.

## 5. Conclusions

The present research shows that the quantification of OBCFA contents in IMF would suffice to separate meat samples according to the NDF to starch ratio of the diet fed to fattening lambs by means of LDA. The odd/anteiso and iso/(anteiso+odd) FA ratios showed negative and positive linear responses, respectively, to the NDF to starch ratio of the diet. A high odd/anteiso FA ratio along a low iso/(anteiso+odd) FA ratio in lamb meat would indicate a feeding regime rich in starch. Conversely, a low odd/anteiso FA ratio along a high iso/(anteiso+odd) FA ratio in lamb meat would indicate a feeding regime rich in NDF. Further research along these lines is recommended for ascertaining the validity of OBCFA contents in meat to authenticate the feeding background of lambs entering the meat chain supply.

## Figures and Tables

**Table 1:** Minimum (Min), maximum (Max) and average contents (mean ± standard deviation, g per 100 g of total fat) of several fatty acid groups in Longissimus muscle from lambs fed the experimental treatments.

Treatments ^1^									
Fatty Acids	CON			CAM			FIB		
	Mean	Min	Max	Mean	Min	Max	Mean	Min	Max
**Total saturated**	32.6 ± 1.76	29.2	34.8	33.3 ± 1.58	30.9	35.3	31.1 ± 2.04	27.1	33.7
**Odd**	2.99 ± 0.57	2.08	3.90	2.66 ± 0.39	2.25	3.25	1.68 ± 0.22	1.42	2.12
**Iso**	0.70 ± 0.09	0.56	0.83	0.59 ± 0.07	0.48	0.65	0.65 ± 0.06	0.54	0.75
**Anteiso**	0.37 ± 0.06	0.30	0.45	0.54 ± 0.07	0.42	0.66	0.55 ± 0.09	0.43	0.76
**Monounsaturated**	36.1 ± 2.56	31.6	41.5	40.1 ± 2.42	37.3	44.7	38.1 ± 2.59	35.6	43.5
**Trans 18:1**	4.47 ± 1.31	2.14	6.18	6.49 ± 0.74	5.70	8.08	8.86 ± 1.38	6.80	11,0
**Polyunsaturated**	17.3 ± 1.30	14.7	18.8	15.4 ± 1.81	12.1	17.9	18.3 ± 2.60	14.1	21.2
**CLA**	0.46 ± 0.07	0.36	0.62	0.57 ± 0.07	0.44	0.64	1.07 ± 0.24	0.75	1.51

^1^ CON: typical commercial concentrate rich in starch and based on cereals and soybean meal. CAM: typical commercial concentrate which replaces 50% of soybean meal with camelina meal. FIB: concentrate rich in neutral detergent fiber based on fibrous by-products and not including cereals or soybean meal.

**Table 2:** Canonical discriminant analysis results.

	Standardized Canonical Coefficients		Canonical Structure	
	DF1 ^1^	DF2	DF1	DF2
**Odd FA ^2^**	1.00	−0.47	0.81	−0.56
**Iso FA**	0.40	0.60	0.36	0.76
**Anteiso FA**	−0.85	−0.34	−0.76	−0.46
**Eigenvalues**	5.37	0.54		
**Variance explained (%)**	90.92	9.08		
**Bartlett test**	*p* < 0.001	*p* < 0.01		
**Canonical correlation**	0.92	0.59		
**Wilk’s lambda test**	*p <* 0.001	*p <* 0.01		
**Centroids ^3^**				
**CON**	2.88	0.37		
**CAM**	−0.43	−0.97		
**FIB**	−2.45	0.61		

^1^ DF: discriminant function. ^2^ FA: fatty acids. ^3^ CON: typical commercial concentrate rich in starch and based on cereals and soybean meal. CAM: typical commercial concentrate which replaces 50% of soybean meal with camelina meal. FIB: concentrate rich in neutral detergent fiber based on fibrous by-products and not including cereals or soybean meal.

**Table 3:** Fisher’s classification functions.

	Treatments ^1^		
	CON	CAM	FIB
**Intersection**	−97.266	−79.860	−75.070
**Odd FA ^2^**	23.388	16.984	10.411
**Iso FA**	168.825	139.528	141.459
**Anteiso FA**	17.119	59.838	75.023

^1^ CON: typical commercial concentrate rich in starch and based on cereals and soybean meal. CAM: typical commercial concentrate which replaces 50% of soybean meal with camelina meal. FIB: concentrate rich in neutral detergent fiber based on fibrous by-products and not including cereals or soybean meal. ^2^ FA: fatty acids.

**Table 4:** Mean separation analysis for the ratios of odd- and branched-chain fatty acids with discriminating ability in the discriminant functions (DF) 1 and 2.

	Treatments ^1^			
	CON (0.31)	CAM (0.40)	FIB (1.54)	SEM ^3^
**DF1: Odd FA/Anteiso FA ^2^**	8.15 ^a^	4.93 ^b^	3.12 ^c^	0.419
**DF2: Iso FA/(Anteiso FA+Odd FA)**	0.22 ^b^	0.19 ^b^	0.29 ^a^	0.011

^1^ CON: typical commercial concentrate rich in starch and based on cereals and soybean meal. CAM: typical commercial concentrate which replaces 50% of soybean meal with camelina meal. FIB: concentrate rich in neutral detergent fiber based on fibrous by-products and not including cereals or soybean meal. Values in parenthesis under each treatment are the neutral detergent fiber (NDF) to starch ratio of the respective concentrate. ^2^ FA: fatty acids. ^3^ SEM: Standard Error of the Mean. ^a,b,c^ Within a row, means without a common superscript letter are significantly different according to Tukey′s test at *p* < 0.05.

## Data Availability

The data presented in this study are available in the article.
